# Person-centred care in the context of higher education – a discourse analysis based on interviews with programme directors

**DOI:** 10.1186/s12909-024-05885-2

**Published:** 2024-08-13

**Authors:** A Jonnergård, I Björkman, E Forsgren, C Feldthusen, M Lundberg, C Wallengren

**Affiliations:** 1 University of Gothenburg Centre for Person-Centred Care (GPCC), Sahlgrenska Academy, University of Gothenburg, Gothenburg, Sweden; 2 Institute of Health and Care Sciences, Sahlgrenska Academy, University of Gothenburg, Gothenburg, Sweden; 3 Department of Health Promoting Science, Sophiahemmet University, Stockholm, Sweden

**Keywords:** National study programmes, Health care professionals, Higher education institution, Interview study, Teaching, Students, Interprofessional Education

## Abstract

**Background:**

As person centred care (PCC) is being implemented globally, higher educational institutions (HEI) have begun to play a crucial part in enabling this transition. In Sweden, however, the delivery of PCC is inconsistently implemented in medicine, nursing, occupational therapy, and physiotherapy study programmes. This inconsistency is partly the result of a lack of a national strategy across HEI. Program directors are responsible for the PCC content of their programs, so their views influence how PCC is taught. Using interviews with programme directors in higher education, we aim to deepen the understanding of the preconditions needed to implement PCC by exploring discourses and identifying subject positions of how PCC is taught and learned.

**Methods:**

We performed a discourse analysis based on interviews with program directors in the above-mentioned national study programmes. A discourse can be seen as a struggle over identity. The subject position – i.e., discourses designate positions for persons to occupy as subjects – guided our analysis and identification of the subject positions of the teacher and the student in teaching and learning PCC.

**Results:**

This study unfolded in two main antagonistic aspects with respect to teaching and learning PCC, resulting in four subject positions for the teacher and four corresponding subject positions for the students. First, the teacher and student were given a subject position as change agents towards a more egalitarian healthcare and were assigned a subject position to cope with a practical reality they could not change. Second, the teacher and student were assigned a subject position that embodied profession-specific identities, navigating and valuing these boundaries. Simultaneously, both teachers and students assumed a subject position that required interprofessional interaction and co-creation for teaching and learning PCC.

**Conclusion:**

This study demonstrates the discursive tension surrounding the implementation of PCC in HEI, and the findings can serve as a basis for creating future relevant and high-quality learning activities. The process of negotiating diverse and co-existing perspectives as well as building interprofessional trust when incorporating PCC into higher education is essential and requires further exploration.

## Background

As the world faces comprehensive health challenges, the World Health Organization (WHO) calls for a fundamental paradigm shift in the way healthcare is funded, managed, and delivered [1]. Such a paradigm shift could come about by adopting person-centred care (PCC) in integrated health services [2]. Adopting PCC means moving from fragmented care to creating an equitable and sustainable health system on all levels – micro, meso, and macro [2]. This kind of change requires collaborative partnerships between stakeholders on all these levels [1–3]. Building on different frameworks, PCC emphasises the co-creation of health service in partnership with patients, their families, and health care professionals [4, 5].

Implementing PCC into different healthcare contexts requires professional training. Education focused on PCC has been seen as an essential aspect of its successful use in clinical settings [6]. In addition, effective PCC requires patients, relatives, and health care professionals across organisations to collaborate as team members [7]. The WHO suggests that higher education students from different healthcare education programs receive their clinical training and therefore their PCC education together [1].

Higher educational institutions (HEI) have a responsibility to educate future health care professionals and to teach and examine students in alignment with societal developments [8]. Therefore, HEI are important facilitators in enabling the transition to PCC. There are, however, great variations in how far the implementation of PCC in higher education has come. PCC is emerging in different healthcare educational programs on various levels, stretching from being briefly and variably visible in local course syllabuses, elective courses, and specific modules [9–11] to more systematic approaches and initiatives in developing PCC frameworks and curricula [12–15].

This study takes place in Sweden within a larger project that focused on PCC in Swedish national study programs. Recently, we found within the Swedish context that PCC could only be identified in local program syllabuses and local course syllabuses, suggesting a bottom-up driven process rather than a national implementation process. In addition, a variety of terms were used to describe the content related to PCC, potentially reflecting a variety of perspectives in HEI regarding the meaning of PCC [16]. These results are similar to findings in other studies that disclose variations in terms and definitions used synonymously and different uses of language describing PCC in curricula [9, 10]. Since program directors are responsible for the content of study programs in Sweden [17], we conducted follow-up interviews with the programme directors of national study programmes in Sweden [18]. This follow-up study outlined the current state of PCC implementation in HEIs and revealed ambiguous and uncertain perceptions of PCC’s meaning and value. The participants also related that PCC, a complex teaching and learning context, requires teachers to consider the students’ own resources and personhood so they can better understand and ultimately implement PCC [18]. Several studies across different educational contexts emphasise the relational and student-centred aspects of teaching and learning PCC. In medical education, dialogic teaching is employed to enhance person-centredness, highlighting the challenge of integrating teaching and learning skills not typically included in curricula [19]. In nursing education, using language that promotes equality between teacher and student is critical in creating a more person-centred curriculum [20]. Furthermore, a study in nursing education showed that the teacher’s role and skills were key for proactive student engagement and learning PCC [21]. Curricula grounded in embodied, person-centred approaches have been suggested to improve student collaboration with patients in a physiotherapy context and deserve further exploration [22].

However, the best strategies for implementing and teaching PCC in HEI have not been identified [10, 11, 14]. Moreover, many have requested that a national initiative and strategies be developed for PCC, including developing curricula for all health care professionals [11, 14–16].

### Discourse and subject positions

The theoretical assumption made when referring to discourse is that access to reality is through language [23]. The main concern in discourse is not about finding the knowledge of ‘true nature’ but to uncover the processes that people engage through discourse [24]. Language is not neutral as it plays an active role in constructing knowledge and therefore identity leads to different understandings of the world. As a result, social actions and social constructions of knowledge have social consequences [23]. Moreover, discourses assign positions for people to occupy – i.e., subject positions [25]. This subject is not seen as an autonomous and sovereign entity that is the origin of social relations. Rather, subject positions are always constructed as a discursive position linked to certain expectations regarding what one can say or how one can act, creating and limiting possibilities for actions [23, 25]. The subject is also fragmented as a person can occupy different positions in different discourses simultaneously, often intertwined and engaged in struggle [23, 25].

Teaching and learning PCC requires advanced understanding of PCC in HEI. To better understand the current preconditions for implementation, we explore discourses of teaching and learning PCC and identify constructions of the teacher and student. Specifically, this study illuminates the subject positions within the discourses for the teacher and student in the context of higher education in Sweden. This exploration was based on interviews with program directors of national study programs in medicine, nursing, occupational therapy, and physiotherapy.

## Method and study design

The study design, a secondary analysis of qualitative research data [26], adds to the knowledge gained from our previous interview study with programme directors, which identified facilitators and barriers for implementation of PCC into HEI [18]. In this previous study, the interviews were analysed using content analysis in an abductive approach applying the Consolidated Framework for Implementation Research (CFIR theoretical framework) for interpretation [27]. During the initial analysis of data in the previous study, there was a consensus in the group that the data also revealed statements addressing the ambiguity and conflicting utterances about the teacher and student while learning PCC. This was only briefly touched upon in that paper. To address and deepen the remaining issues, we settled on an approach that could illuminate these tensions. Therefore, we performed a separate analysis using discourse methodology, highlighting the interactive discursive processes revealed in the spoken words in the data. Examining the subject positions of the teacher and student creates an opportunity to identify the actions available and to understand what is enabled and constrained within different discursive structures [23].

### Participants

Programme directors (*n* = 48) from all national study programmes in medicine, nursing, occupational therapy, and physiotherapy were invited to participate via mail. We followed up non-responses with an additional e-mail. Programme directors (*N* = 19) from national study programs representing medicine (*n* = 4), nursing (*n* = 7), occupational therapy (*n* = 5), and physiotherapy (n = 3) participated in the study. The program directors had experience teaching in higher education and clinical practice. Different educational programs, geographical areas, universities, and university colleges are represented [18].

### Data collection

Individual semi-structured interviews were performed by AJ, IB, and IKL via Zoom videoconferencing software or telephone and lasted between 22 and 67 min. A semi-structured interview guide was used and validated through three pilot interviews. The guide was revised allowing for greater openness to explore the informant’s perspectives. The interviews addressed views and experiences implementing PCC, concepts used in relation to PCC, and how teaching and learning PCC could be achieved. There were continuous discussions in the research group to ensure richness in the interviews in relation to the aim. The interviews were transcribed verbatim by professional transcribers, resulting in 323 double-spaced pages. They were stored in compliance with ethical guidelines and Swedish law [28].

## Secondary analysis

### Discourse theory and method

In this study, we conducted a discourse analysis inspired by Lauclau and Mouffe [25] and by Jorgenson and Phillips [23]. Lauclau and Mouffe’s discourse theory analyses the conflict over identity – referred to as antagonism. Antagonism could be seen as the tension that arises when different identities prevent each other from manifesting [23, 25]. Since no discourse can be established completely, there is always room for struggles with other discourses that have different definitions of reality and different guidelines for social action [23]. Identity means identification with a subject position in a discursive structure [25]. The concepts of antagonism and subject position shape our analysis [23, 25]. To guide our analysis further, we also used the analytical strategy of exaggerating and comparing details [23].

### Data analysis

First, the authors independently read the interviews several times to get an overall picture of the material and to identify preliminary discourses. Then, all the authors discussed and compared the interviews. In this step, larger and smaller discourses were identified. Next, we identified several issues the discourses revealed – e.g., the subject positions assigned to the teacher and the student. We marked sequences and statements and looked for patterns. We noticed conflicting statements and tensions. The concept of antagonism and the notion of subject position helped delineate specific discourses as well as subjects within the identified discourses [23]. Finally, using an iterative analytical process, we formed some preliminary findings [23]. The interviews were compared to each other searching for underlying assumptions in statements and making them explicit. This process was conducted to establish elements that supported conflicts of interpretation and features, to illuminate our own preconceptions in the analysis, and to work with the material in a comprehensive way [23]. Data analysis involved an iterative process where findings were continually discussed, looking for disagreements within and between different discourses and subject positions and comparing the interviews and the interpretations of the material as a whole.

During the analysis, we were deliberately mindful of our own pre-understandings and identities in relation to the research topic and how these might affect our analysis. All the authors are involved in teaching as well as researching PCC. As researchers, we are embedded in the discourses and shared assumptions that we are aiming to reveal. Different experiences in the research group and continuous critical discussions helped us consider our preconceptions in a reflexive way [23]. We strived to approach the material anew, maintaining openness in order to be aware and critical of the evolving understanding throughout the process. Transferability needs to be interpreted by the reader [29]. To present the analysis in a transparent way, we used quotations that the reader can use to test the claims made [25].

Data analysis was mainly conducted by AJ in collaboration with IB and EF but involving all authors in different stages of the process. All authors are connected to Gothenburg Centre for Person-Centred Care (GPCC) and from three different professions (nursing, physiotherapy, or speech language therapy) and teach at national study programmes in nursing, medicine, or physiotherapy.

### Ethics

An ethical application for the study was submitted to the Swedish Ethical Review Authority, who decided that no ethical approval was needed for the study but gave an advisory opinion, having no ethical objection of the performance of the study (reference number 2020–05677). The study was conducted in line with the Helsinki Declaration [28]. All participants were given oral and written information about the study aim individually, and their right to withdraw at any time. We obtained informed written consent by mail and verbal consent from all participants was reinsured in the beginning of the interviews to take part in the study.

## Result

**Fig. 1:**
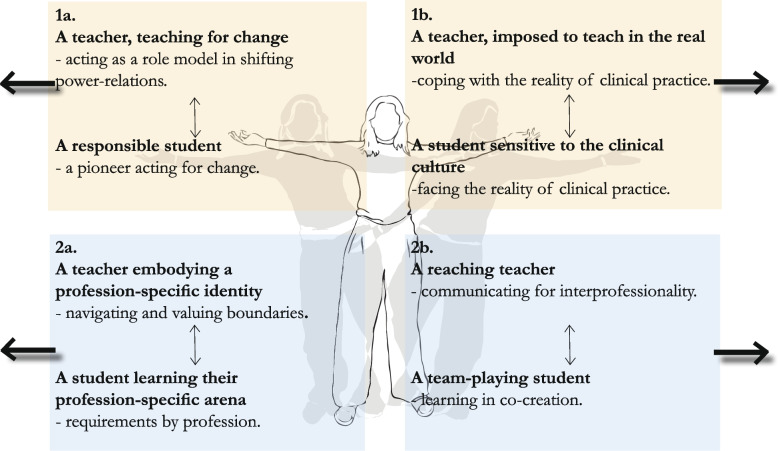
Subject positions of the teacher and the student in teaching and learning PCC

Based on programme directors’ interviews, this study unfolded in two main antagonistic aspects with respect to teaching and learning PCC. This resulted in four subject positions for the teacher and four corresponding subject positions for the students, interpreted from the perspective of programme directors in HEI (Fig. 1). The first antagonistic aspect concerns how teaching and learning PCC was seen as connected to a societal trend towards a more egalitarian healthcare system. The teacher was given a subject position as a role model in teaching for this change and the student as a pioneer in transforming the healthcare system. On the other hand, as teaching and learning PCC were seen within the current healthcare system as an unattainable position, the teacher and student were required to cope with and to be prepared for this reality. The second antagonistic aspect deals with how PCC is negotiated from a profession-specific perspective. The different professions, embodied as profession-specific identities, required the teacher and student to navigate and value these boundaries when teaching and learning PCC per the requirements of their profession. On the other hand, PCC includes a subject position where the teacher and student, through the teaching and learning process, co-create and bridge communication between professionals. These conflicting and intertwined subject positions pull on the teacher and student often in opposite directions when teaching and learning PCC in the context of higher education.

### 1a. A teacher, teaching for change – acting as a role model in shifting power relations

The interviews revealed that PCC was an ongoing societal trend forming a more egalitarian healthcare system that equalised power in relation to the patient. This equalised power was considered as important as the actual tasks of teaching PCC within a healthcare system. The teacher in the subject position was interpreted as someone acting as a change agent by shifting power relations towards the student. The interviews revealed that the participants see a highly self-reflective teacher as someone who sees the potential of person-centred work with the student and who realises a need to work in a person-centred manner with the student and therefore contributing to a more egalitarian healthcare education in teaching PCC. With respect to their students, the teacher needs to embody a person-centred approach. Thus, the learning situation itself was seen as an opportunity to demonstrate how PCC works as it acts out the teaching in the educational setting as the students would do with a patient in a healthcare context, a view evident in the following quotation:

We have to practice what we preach and try to see what has happened now. And try to learn, so that maybe we can do it differently, so that we don’t end up in this situation again. […] that it doesn’t become finger-wagging and that we are the ones who decide, and … paternalistic. (Programme director of a nursing programme)

### A responsible student – a pioneer acting for change

The student in the subject position was interpreted as a pioneer in making healthcare practices more person-centred. The students act according to their own agency and belief, building on their own understanding of the patient. The students in this subject position are given the opportunity to reflect, to be seen, and to take responsibility for their action, enabling them to lead themselves and learn on their own. The students were interpreted as independent agents who take responsibility for their knowledge acquisition, creating trust in themselves and with the teacher, who they assume will provide effective feedback. From this subject position, the students are educated to transform healthcare and their own roles within healthcare. The student’s own learning process is seen as the driving force for implementing a more person-centred approach, a development that needs to be attended and supported:

We’re not just conducting an education here and now; you are pioneers. It’s you [the student] who will improve our ability to actually implement this [person-centredness] out there when the time is ripe. (Programme director of nursing programme)

### 1b. A teacher imposed to teach in the real world—coping with the reality of clinical practice

The current situation within healthcare practices is stretched to its limits, and teaching and learning PCC remains as an unattainable position for the students in clinical practice. The programme directors referred to the clinical practice as tough, task-oriented, and not very person-centred and therefore provides limited learning opportunities in PCC for the students. The teacher in the subject position needs to protect their students by helping them cope with this discrepancy between the theoretical education and the reality of clinical practice. The programme directors referred to clinical practice as the most significant learning context, offering the most impactful learning experiences. The academic teacher from this subject position can only guide and have limited control over the theoretical education, whereas the real learning about PCC is shaped in the clinical practice. What is translated and taught about PCC depends on individual clinical supervisors as well as the context. Therefore, teaching PCC must be negotiated as it should address the pressure and the expectations placed on the students when they enter the workforce:

In many healthcare settings, nurses are under tremendous pressure […] recent nurse graduates are beginning to discuss stressful ethical and moral situations as they step into a tough reality. (Programme director of nursing programme)

### A student sensitive to the clinical culture – facing the reality of clinical practice

The programme directors pointed out how learning PCC meant incorporating a whole approach centred around the patient. Program directors questioned what this approach means for the students’ acquisition of knowledge and skills. In this subject position, the students were characterised as vulnerable to the prevailing structures and unable to defend themselves against the culture they encounter in clinical practice. At the same time, the students were seen as having different abilities with respect to learning a person-centred approach. That is, the students were seen as tending to adopt a more normative approach and conform to the traditional culture of clinical practice:

But then I see them [students] later as house officers, and they seem to have lost it [a person-centred approach], in my opinion. [When] they have started their time in the medicine and surgery departments [laughs] … they’ve been shuffling patients through the emergency department [unable to practice a person-centred approach to healthcare]. (Programme director of a medical programme)

The student’s focus is initially task oriented – i.e., they need to master their professional skills rather than work on person-centred skills. The programme directors positioned the students as people who do not fully grasp the broader perspective that a PCC approach requires, being engaged in an ongoing learning process, and initially immature in their professional practice:

It’s part of the development process, to a certain extent difficulties in actually handing over responsibility, in the meeting with our patients or individuals who in some way … may feel a bit threatening. That some students feel they need to wear armour … to feel secure in their work. (Programme Director of occupational therapy programme)

### 2a. A teacher embodying a profession-specific identity – navigating and valuing boundaries

A pattern was seen in how PCC was negotiated in relation to profession-specific historicity – i.e., different knowledge traditions shaped how teaching and learning PCC were viewed. As the teacher embodied these points rooted in different ontologies and epistemologies, the teaching and learning of PCC were negotiated from these different perspectives. For the medical teachers, experiences with patient-centred consultation were seen as the most in line with PCC. They referred to PCC as an approach that respected patient autonomy, patient co-determination, and the Swedish Patient Act, which were also mentioned by the physiotherapists. The physiotherapist teachers also embody the concept of movement, bodily interactions, and the therapeutic alliance as forming their teaching in relation to PCC. The nursing teachers were interpreted as carrying narratives strongly influenced by various philosophical traditions, named theorists, and underlying philosophical views of human nature. The occupational therapist teachers held the client in the centre and relied on different models to inform their view of PCC. Embodying these perspectives, the teacher is from this subject position someone who navigates and values their boundaries in relation to teaching PCC:

We're uncertain when this concept of person-centred care emerged. Is this something different? Is it different from working with client-centred approaches? … We weren’t prepared to work to implement it. Instead, we continued working with what has always been our focus that is, the client always being in focus, always involving, engaging, enabling participation, negotiating rehabilitation plans, and so on. (Programme director of occupational therapy)

### A student learning their profession-specific arena – requirements by profession

The students were positioned as learning to act within their profession-specific arena, influenced by the requirements of the profession. Within this subject position, the students learning PCC negotiate the specific expectations and demands placed on them by their profession. For example, the narratives revealed that medical students need to navigate nuanced ethical situations such as the desire from patients to terminate treatments. A physiotherapist student also needs to handle great responsibility, often working independently and being the first point of contact with a patient seeking care. A nursing student is seen as imbued with the fundamental principles of care where PCC follows naturally. Instead, there is a need to pinpoint nursing a bit more as their field of knowledge. The occupational therapist students hold their occupation as a unique contribution in healthcare. These requirements by profession concerning students’ development and learning their profession-specific arena were interpreted as delineating features in learning PCC. These profession-specific arenas were related to the process of becoming a professional and to how the students distinguish their profession from other healthcare professions and within different contexts:

I believe that there are variations in how one perceives this [person-centredness], which can be influenced by everything from tradition within their discipline to factors I mentioned earlier, like the type of activity. (Programme director in a medical programme)

### 2b. A reaching teacher – communicating for inter-professionality

Teaching PCC was seen as an opening for interprofessional collaboration during education. This worked in both directions – one’s own profession and other professions. The teacher was placed in a subject position as someone who interacts with other professions. The program directors wanted to create a communicative space between professionals in the teaching of PCC. The teacher was then seen as someone who could begin the discussion about the importance of communicating and collaborating with other health care professionals. However, the interviews reveal that something more is needed to help teachers promote interprofessional collaboration:

So, I think, an individual within healthcare is someone who interacts with many professions and, as such, I believe it’s an advantage if we use the same terminology. That there is a conceptual framework that is similar, regardless of whether you meet an occupational therapist, a physician, a physiotherapist, a nurse or whatever it may be. (Programme director in a physiotherapy programme).

### A team-playing student – learning in co-creation

The programme directors positioned the students as part of a team, co-creating with their teachers what it means to be an advocate and practitioner of PCC. In this subject position, the student was interpreted as someone who acknowledges that the understanding of their own profession is to be understood in relation to others with the starting point of the patient as a member of the healthcare team, with different professions listening to the patient in different ways. Therefore, there is an opportunity for students to learn from each other about PCC. For the students to learn PCC, the interprofessional perspective should be part of their education:

In larger cities, we have the luxury of having multiple programs at the same university. In such cases, we can create clinical training department to work interprofessionally and person-centred. However, I think it is crucial to invest in this [interprofessional learning] because it can also become a kind of silo, where one works with one profession at a time. (Programme director of nursing programme)

## Discussion

We explored discourses and identified subject positions of teaching and learning PCC from the perspective of programme directors in HEI. Although a discursive pattern emerged depicting teaching and learning PCC as a wave of change, it also remained as an unattainable position under unchangeable circumstances. Furthermore, teaching and learning PCC was negotiated within profession-specific boundaries while simultaneously reaching out to have interprofessional engagement. Our results highlight the need to navigate the opposing and intertwined expectations placed on both teacher and student, without collapsing into the perception that either position is inherently more person-centred in teaching or learning PCC.

From the standpoint of change, fulfilling the leadership function means being engaged in leadership [30]. In HEI, programme directors are engaged in leadership as academic leaders and key decisions makers in the implementation of change [27]. Our results show that the directors constantly mirrored the academic setting with clinical practice as the subject positions while being pulled in different directions with the teaching and learning placed in the gap between clinical practice responsibilities. The core of PCC is built on an ethical understanding acknowledging the patient as a person with agency as well as recognising the underlying vulnerability in balancing the autonomy of the person [4, 31]. The equal partnership is at the same time asymmetrical as it depends on health care practitioners as gatekeepers [4]. Similarly, our results suggest that both the academic teachers and the clinical supervisors are the gatekeepers in teaching students PCC. Consistent with the findings from our results, students have reported a lack of learning opportunities in PCC in clinical settings due to busyness, unawareness of PCC, and a lack of role models and trained supervisors [32, 33]. Handling this gap is something HEI and programme directors constantly need to deal with and participate in bringing about change. Furthermore, the teacher was interpreted in a subject position of someone who realises a need to work in a person-centred manner with their students and embody the educational context as a reflection of a healthcare situation. Earlier research on implementation of PCC in HEI has pointed out dialogic teaching to promote students’ reflections and address ethical dilemmas in practice [34]. Here, the students were given the subject position as promoters of a more person-centred health care. However, they were influenced by the current situation within healthcare practices, established understandings, and the complexity associated with PCC healthcare, and therefore remained vulnerable—an issue that needs attention. In line with our results, earlier research points out that it takes time to learn to balance the complexity of one’s professional role – i.e., having a person-centred focus [35].

The profession-specific identity was a discursive pattern strongly evident in the data. The subject position of the teacher embodying different ontological and epistemological viewpoints created different starting points, unfolding as navigating the boundaries to teaching and learning PCC from one’s specific professional context. In implementing PCC in HEI, it is important to understand the profession-specific responsibilities and knowledge based on how professionals work together and work separately as this impacts how they will understand and teach PCC. According to earlier research, these dimensions have been overlooked when implementing PCC into clinical practice [36]. For example, a study of applying a PCC framework in collaborative planning in a clinical context emphasised how professionals lacked knowledge of each other’s roles, practiced domain thinking, and struggled with power, trust, and responsibility [37]. Nevertheless, our results suggest that effective teaching and learning PCC requires inter-professional openness with the potential to increase the understanding of each other’s professions and the shared approach in co-creation with the patient. Thus, learning PCC provides the opportunity to create a communicative space to facilitate the learning process, a more dynamic hierarchy, and a more integrated team approach with students. Studies on interprofessional education have shown that performing team-based patient encounters leads to new insights and views of the patients, their health issues, and enhanced collaboration between students [38]. Another study showed a shift in students when their awareness of the tension between professional and person-centred focus increased and promoted the learning to become an interprofessional [39]. Perhaps this issue of understanding different epistemologies and knowledge cultures at a deeper level needs to be seriously considered when implementing PCC in HEI. A challenge identified in implementing PCC in clinical practice has been attributed to the lack of discussion surrounding professional boundaries, interprofessional relations, and discipline-specific knowledge. This silence has been recognised as having implications for improving teamwork and integrating PCC [36]. Illuminating, describing, as well as questioning these subject positions can help breach this silence. Once this silence is breached, HEI can develop the conditions for collaboration and interprofessional trust and understanding in teaching and learning PCC, enabling the transition toward a PCC healthcare education.

## Methodological considerations and limitations

In qualitative research, the whole research process needs to be continuously and critically scrutinised regarding the study’s research findings concerning preconceptions, reflexivity, and transferability [29]. All the authors of this paper research PCC and are involved in teaching – nursing, physiotherapy, or medical education. As a result, we are embedded in these discourses and therefore have preconceptions about the phenomena. Although these preconceptions may have challenged our ability to deconstruct the most elusive discourses, they could have deepened the understanding of the discourses involved. A criticism of discourse analysis is that it operates on a superficial level; however, discourses are constitutive rather than definitive – i.e., a discourse should ultimately be seen as only one discourse among others [23]. Lauclau and Mouffe are criticised for overestimating the possibility for change as they seem to ignore the important structural conditions of class, ethnicity, and gender [23]. In our study, the interpreted subject positions could be seen as simplified as not everyone has the same access to the same subject positions. This limitation highlights an underlying complexity in the assignment and accessibility of subject positions, pointing to the challenges in creating new opportunities for action [23]. Interviews with programme directors were used as empirical data. These data are viewed as constructed between social subjects using the interviewers’ own subjectivity and therefore one must reflect on the power relations in the interview situation [40]. The interviews were performed in an academic setting with a focus on implementing PCC in HEI. Considering this as a secondary analysis, there is a risk that there are dimensions that should have been explored further but are not fully grasped in the interviews from the perspective and purpose of this study. Reporting these data, however, was a way of disclosing the entire material as transparently as possible.

## Conclusion

This study demonstrates the discursive tension in teaching and learning PCC in HEI from the perspectives of programme directors. The findings highlight a complexity in collaboration and co-creation across academic and clinical contexts to create effective PCC healthcare education fostering students’ professional identities and enabling them to become trusted team players. This study is included within a larger project attempting to co-create an educational module to support the teaching and learning of PCC in HEI. Illuminating the current complexity of social practice can serve as a basis for creating future relevant and high-quality learning activities. In addition, the process of building interprofessional trust and negotiating diverse and co-existing perspectives when incorporating PCC into higher education are crucial areas for further investigation.

## Data Availability

The datasets analysed during the current study are available from the corresponding author on reasonable request.

## Abbreviations

CFIR: Consolidated Framework for Implementation Research
GPCC: University of Gothenburg Centre for Person-Centred Care
HEI: Higher Education Institutions
PCC: Person-Centred Care
WHO: World Health Organization
